# Drivers for coping with flood hazards: Beyond the analysis of single cases

**DOI:** 10.4102/jamba.v11i1.678

**Published:** 2019-04-09

**Authors:** Roland A. Balgah, Henry N. Bang, Salliana A. Fondo

**Affiliations:** 1 College of Technology, University of Bamenda, Bamenda, Cameroon; 2 Disaster Management Centre, Bournemouth University, Dorset, United Kingdom; 3 School of Life Sciences, Technical University of Munich, Munich, Germany

**Keywords:** disaster risk management, natural hazards, multiple cases, coping strategies, floods, local development

## Abstract

Flood risks continue to pose serious threats to developing countries with dire ramifications for livelihoods. Yet, contemporary research on determinants for coping with flood hazards is driven mostly by individual cases with less effort to systematically identify coping strategies across multiple floods. This research analyses potential determinants of coping strategies to flooding across multiple floods using two case studies in Cameroon. Via empirical research and qualitative or descriptive statistical analysis, the research investigated how human, social, and economic or financial variables influence household coping decisions across the two flood sites. Results suggest a great influence of social and human capital on household decisions to adopt specific coping strategies and that over 80% of flood victims in both study sites applied post-flood informal coping strategies. Analysis also shows significant inconsistencies with human capital variables, which reveal that coping determinants can be quite different even for floods occurring in the same agroecological zone. The findings also reveal that economic and financial capital has little influence on flood victims’ coping decisions, contrary to popular contentions in the literature. The results of this study have implications for research and policy implementation on flood-induced coping strategies in developing countries.

## Introduction

The consequences of upsurging natural hazards and disasters are now a terrifying reality with dire ramifications for natural, social, human, financial and physical capital. Yet, managing their impacts remains one of the greatest contemporary challenges in flood-prone countries. Natural hazards took away 1.2 million lives over the decade between 2002 and 2012, affecting 268 million people and causing economic damages worth $1.7 trillion (UNISDR 2015). Indeed, the number of people affected by natural disasters worldwide is on the rise with annual economic losses between $250 million and $300 million (UNISDR 2015; World Disaster Report 2015). In 2016, global natural disasters caused economic losses of US$ 210 billion – 21% above the 16-year average of $174 billion. Flooding, earthquakes and severe weather were the topmost hazards, which together, produced 70% of the economic losses in 2016 (Aon 2017).

Worst still, natural disasters are having a serious toll on the world’s poorest people, exacerbated by increasing hazard complexity. In view of the increasing fatalities and economic costs of natural hazards and disasters, the need for reducing hazard risks and enhancing appropriate coping and resilience strategies cannot be overemphasised (Serre et al. 2016). Therefore, the implementation of effective disaster management strategies is vital to governments if they have to achieve the Sustainable Development Goals (Clark 2015). This is even more crucial for many developing countries, where state and market institutions for effective disaster management (such as disaster relief and insurance schemes, respectively) are often missing or only exist suboptimally (Edoun, Balgah & Mbohwa 2015). In such countries, efforts towards achieving the Sustainable Development Goals can be quickly wiped out by natural hazards and disasters. Curbing such losses will enhance sustainable development in such countries.

**FIGURE 1:**
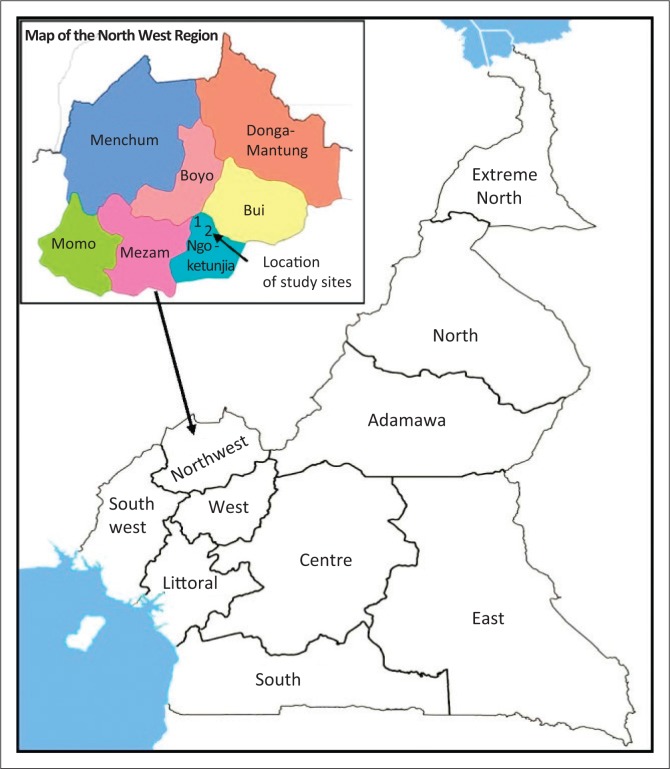
Cameroon map showing the study sites (1 – Babessi-Flood I; 2 – Baba I-Flood II) in Ngoketunjia division of the Northwest Region.

One limitation in the management of hazards and disasters in developing countries is the frequent absence of early warning or preventive systems, including mitigation, coping, adaptation and resilient measures. Furthermore, institutions for hazard management in many developing countries are very weak (Balgah et al. 2016; Bang 2014). Consequently, large parts of Africa and Asia will continue to experience pervasive devastation from increasing frequency of hazardous events, further impeding economic growth in these continents (Edoun et al. 2015). Reversing such negative paradigms requires an understanding of vulnerabilities and impediments to victims’ coping strategies to climate-related hazards, including identifying determinants that are robust across space and time (Berman, Quinn & Paavola 2014; Speight, Hall & Kilsby 2017). This paper contributes in this direction, by examining the drivers for coping decisions for two separate floods in Cameroon (see Figure 1).

The next section concisely revisits the natural hazards literature, emphasising the key role of floods. Section two has a succinct overview of the determinants for household coping decisions based on single case studies. Section three presents the materials and methods implored in the empirical examples. Empirical results are presented and discussed in section four before concluding the paper.

## Literature review

### Natural hazards: Generalities

The global frequency and severity of natural hazards is causing great economic losses, socio-political instability and increasing vulnerability to poverty whilst manmade hazardous events are exacerbating the effects of these hazards. Moreover, there is considerable uncertainty on current and future natural hazard trends. However, predictive modelling suggests that future occurrence of natural hazards will generally result from climate oscillations (IPCC 2014).

Hydro-meteorological hazards, mainly droughts and floods, account for almost 90% of global natural hazards (IPCC 2014). The bulk of the estimated total economic loss of US$3.8 trillion from natural hazards in the next few decades will emanate from developing countries and 75% of all economic losses and over 60% of total lives lost will continuously affect Africa, particularly sub-Saharan African (SSA) countries where poverty is pervasive (World Bank 2014a). These grave consequences will be further compounded by high levels of exclusion, vulnerability and limited government hazard management capacities (Bang, Miles & Gordon 2017; Edoun et al. 2015).

Floods are the most important global form of natural hazards in contemporary Africa and accounted for almost 83% of all disasters in Africa in 2010, causing an economic loss of $59.2 million in the same year (Guha-Sapir, Hoyois & Below 2013; Ozger 2017). In addition, this situation is set to worsen with climate change (IPCC 2014). Indeed, climate-related disasters that cause flooding are on the rise worldwide and accounted for 87% of natural disasters in 2014, continuing a 20-year-long trend of climate-related disasters (World Disaster Report 2015).

Currently, increasing flood frequency affects assets, entitlements and livelihood security especially for the poor in hazard-affected Africa (Mbereko, Chimbari & Mukaratirwa 2018; Ngwa et al. 2015). The direct effects include forceful displacement, destruction of houses and fixed assets, increased disease prevalence and loss of human lives (Buchenrieder, Mack & Balgah 2017; Dube, Mtapuri & Matunhu 2018; Ntungwe 2015). However, the victims are not always passive in the face of such events. Often, they may apply either adaptive or coping strategies. Whilst adaptive strategies aim at minimising flood risks, coping strategies are employed to deal with the aftermaths (Serre et al. 2016).

Often, hazard victims prefer preventive strategies, but have no option than to adapt in their absence (Berman et al. 2014). Some adaptive and coping measures employ formal and informal instruments. Informal instruments include individual, household or community-based actions whilst formal instruments are mainly market and public interventions. Examples of informal pre-hazard instruments include crop diversification, off-farm employment, informal credit and insurance arrangements, creating buffer stocks or adhering to risk-sharing networks (Fafchamps & Lund 2003:210). Informal post-hazard strategies include buying and selling of real assets, informal borrowing, asset liquidation and consumption smoothing. A common and widely known example of a formal pre-hazard instrument is an insurance premium. Some formal post-hazard instruments include disaster relief, government or non-governmental organisations’ (NGO) support, social capital or networks – community assistance, saving and food stocks and sales of personal assets or livestock if not destroyed (Abid, Schneider & Scheffran 2015; Buchenrieder et al. 2017; Holzmann 2001; Mbereko et al. 2018).

The need to understand how victims make these decisions prior to, during or after natural hazards and under what circumstances they would prefer some actions over others is crucial in enhancing long-term prevention, reduction, coping, adaptation as well as for building resilience in hazard-prone communities. Clearly, this is a herculean task for any one piece of scientific work. With this understanding, this paper makes a modest contribution by analysing the determinants for adopting specific coping strategies by hazard victims, based on two case study floods in Cameroon. This hinges on the contention that determinants, which are robust across space and time, are likely to have stronger policy implications than those identified in single, isolated case studies.

### Flood hazards: A succinct overview of relevance and impacts

Flooding is one of the Earth’s most common, most destructive and most deadly natural hazards and flood damages continue to rise worldwide with dire consequences for natural, social, physical, financial and human capital (Bang et al. 2017). Over 50% of all disasters recorded in 2010, for instance, were of floods origin, representing an increase of over 145%, compared to the mean between 2000 and 2009. In the same year, floods inflicted harm to an estimated 2.6 billion people worldwide, leaving behind direct and indirect economic losses of about $46.9bn (Guha-Sapir et al. 2013). Contemporary flood impacts have serious direct and indirect social, psychological and economic effects on victims – destruction of property, loss of lives and displacement, reduced human dignity, posttraumatic psychological and mental disorders, and increased frequency of diseases (Bang et al. 2017; Ntungwe 2015). Flood effects are often more severe on the very poor in developing countries, mainly because of weak disaster management systems (Bang 2014) and rampant state and market failures to contain flood aftermaths (Ntungwe 2015). Understanding drivers for coping with flood hazards can stimulate flood impact, reducing policy options of relevance across many developing countries.

### Determinants for coping with flood hazards

Flood victims often engage in strategies that can best support them cope with the aftermaths. In developing countries, informal coping strategies are more common because state and market mechanisms are often absent, dysfunctional or unreliable (Berman et al. 2014; Edoun et al. 2015; Holzmann 2001). Nevertheless, when these mechanisms are present, victims would not hesitate to appropriate them, often combining the formal with informal instruments (Balgah, Buchenrieder & Mbue 2012; Balgah, Buchenrieder & Zeller 2015).

Research evidence suggests that socio-economic factors fundamentally determine victims’ decisions to adopt specific flood-coping strategies. Berman et al. (2014), for instance, found out that age, education and household wealth crucially influenced the coping choices for flood victims in Uganda. Older households relied more on social support than younger ones, whilst the more educated households relied largely on own savings to buffer flood effects. However, Boamah et al. (2015) report mixed results in their cross-sectional survey amongst flood victims in Tanzania and Nigeria. Similar to Berman et al. (2014), age and access to economic resources were found to influence the type of coping strategies adopted by victims in Nigeria. This was not the case in Tanzania, where other factors were more important. This inconsistency is probably because of context-specific factors, which were not captured in the research.

Key drivers for coping decisions identified in topical literature include social capital abundance and economic activities (Armah et al. 2010; Balgah et al. 2015; Fafchamps & Lund 2003; Jakiela & Ozier 2012); access to alternative natural resources (Berman et al. 2014; Cervantes-Godoy, Kimura & Anton 2013); past experiences (Boamah et al. 2015; Mbereko et al. 2018); gender and occupation of the household head (Balgah et al. 2016; Mobarak et al. 2012); poverty levels, risk exposure and access to economic resources (Bryan, Chowdhury & Mobarak 2011; Cervantes-Godoy et al. 2013; Holzmann 2001; Meredith et al. 2013); information (a)symmetry (Jensen 2012); risk perceptions, culture and beliefs (Balgah et al. 2015; Wachinger et al. 2013); and the functioning levels of state and market hazard management institutions (Cervantes-Godoy et al. 2013; Holzmann 2001).

For instance, Meredith et al. (2013) reveal that liquidity constraints and high prices negatively affected coping decisions amongst hazard victims in Kenya. Similar findings in Cameroon reveal that very limited finances prevent victims from easily coping and adapting to flood hazards (Ngondjeb 2013). Similarly, Bryan et al. (2011) in a more restricted study demonstrated that access to improved agricultural techniques, educational levels and information asymmetry strongly influenced household flood-coping choices in Bangladesh. Balgah et al. (2015) report the unwillingness of flood victims in rural Cameroon to move to new havens, as existing cultural and belief systems restrict them from living far away from the graves of their beloved ones. Dube et al. (2018) in a broader sense relate poverty to flood risk management in Zimbabwe.

Contemporary research on household coping decisions to floods generally contends that coping decisions are spatially ubiquitous. With the exception of a few studies (see Berman et al. 2014; Boamah et al. 2015; Ozger 2017), knowledge on coping with flood hazards is largely contingent on cross-sectional, individual case-specific studies. Analysing multiple case studies will arguably identify determinants, which are robust over space and time (Speight et al. 2017). Such determinants imbibe greater potentials for policy. This paper embraces this philosophy by analysing two floods cases from Cameroon.

## Materials and methods

### Background of the study area

This study was done in Cameroon, one of the SSA countries highly affected by floods. Its Northwest Region (see Figure 1), the third most populated region in the country (over 1.8 million), has increasingly witnessed flood episodes especially in the last decade, mainly as a consequence of climate variability (Innocent, Bitondo & Balgah 2016). These floods have dire consequences for the predominantly agricultural populations in the region. Indeed, over 75% of the population in Northwest Cameroon depends on subsistence, rain-fed agriculture for their livelihoods (Yengoh 2012). Besides, the poverty rate in the region, estimated at 51%, represents 13% of the total poor in the country (Ambagna, Kane & Oyekale 2012). Two seasons dominate the region, namely the rainy season, spanning from mid-March to the end of October, and the dry season, from November to mid-March (Innocent et al. 2016).

The study area hosts two communities (Babessi and Baba I), located in Ngoketunjia, one of the seven divisions that make up the Northwest Region of Cameroon (see Figure 1), which has been struck in recent years by two independent devastating floods:(1) The Babessi floods of September 09, 2012, (hereafter also called Flood I). This flood resulted in the displacement of 4000 inhabitants from 50 families and complete destruction of 26 homes (Loh 2012). In addition, victims lost over 60% of their livestock and almost 100% of cash held in the household at the time of the floods. Food crops and agricultural lands were greatly damaged, and food consumption at household level dropped from a mean of three to two meals per day, pre- and post-floods, respectively (Balgah et al. 2015). (2) The Baba I flood of September 14, 2015 (hereafter also called Flood II). Baba I is a neighbouring community to Babessi. This flood left around 100 people from 65 families homeless, completely submerging 35 homes and causing enormous economic, agricultural and psychosocial damages in the community (Bruno 2015). Households were reported to have adopted predominantly informal coping strategies such as relying on family support and community solidarity to initially deal with the immediate floods aftermaths (Balgah et al. 2015, 2016). We use these two case study floods to analyse the drivers for household decisions to predominantly adopt informal coping strategies in both floods. A number of factors make this comparison interesting. Firstly, Babessi is host to all of the administrative units under the Babessi Council area. As such, it is more cosmopolitan than Baba I, which is typically rural. Secondly, most of those affected in these two research sites depend on agriculture for their livelihoods (Balgah et al. 2015). However, because of the urban nature of Babessi, it tends to depend on Baba I for a substantial supply of food. Thirdly, there is a 3-year window between the two floods, which could have induced experiential knowledge sharing and disaster preparedness. A key interest here is to identify drivers that are robust over space and time of more relevance to policy than is obtainable from isolated cross-sectional case studies currently dominating the floods literature.

### Methodology and sampling approaches

The choice of the case studies is logically justified from at least four fronts. Firstly, developing countries are hypothesised to be most exposed to the current and future effects of floods (Edoun et al. 2015; IPCC 2014). Therefore, it is important to draw empirical examples from these countries for analysis, where results will be highly relevant for policy. Secondly, floods are amongst the most frequent type of natural shocks worldwide (Guha-Sapir et al. 2013; OECD 2016). Researching on floods would therefore have implications beyond Cameroon, from which the case studies are drawn. Thirdly, Cameroon is one of the countries in SSA greatly affected by floods (Bang et al. 2017; Ngwa et al. 2015). Drawing case studies from this country can potentially have policy implications. Fourthly, although the two case study floods analysed here occurred in different years, they share some similarities, namely, that (1) they are both in the same agroecological zone in Cameroon and (2) the floods occurred in the same month of the year (September). Such a choice minimises the effects of extraneous variables that cannot be accounted for by their search. In addition, the research team had previously collected data on Flood I. This enhanced motivation and created a window of opportunity to apply the same research approach and instruments for Flood I to the Flood II case study, allowing for comparative analysis.

### Data collection and analysis

The data analysed here was collected only from flood victims. Sampling was done at the household level. A structured questionnaire modified following Grotaert et al. (2004) and Zeller et al. (2006) was used in both surveys to allow for comparability. Trained enumerators in both cases collected data less than 2 months after the floods in order to deliberately reduce data unreliability that was likely to accompany long recall periods. Interview and data recording took place at the homestead of the interviewees, allowing the research team to observe some of the effects mentioned during the interview. Seventy-three victims (38 out of 56 for Flood I and 35 out of 65 in Flood II, respectively) representing almost 61% of all the victims participated in the survey. Victims who were not sampled simply refused to participate in the survey or were not in the communities at the time of the survey. Observations and key informant interviews complemented the survey. Collected data were entered and analysed using SPSS (Statistical Package for Social Sciences), version 20.0, and results were validated at 95% confidence interval (α = 0.05). Logistic regressions were performed to identify key drivers for household coping decisions in the two case study floods. Results of the analysis are presented and discussed in the next section.

## Results and discussion

### Descriptive socio-economic characterisation of the sample

Most flood victims in our case studies have only primary school education (71.8% in Flood I and 60% in Flood II, respectively), with almost 70% of the entire sample largely depending on subsistence agriculture for their livelihoods. Furthermore, most victims are married and living together with their spouses (about 77% for Flood I and 80% for Flood II). Table 1 has additional descriptive statistics.

**TABLE 1:** Descriptive statistics of the sampled households by flood incidence.

Variable	Village	Minimum	Maximum	Mean	Standard deviation
Age of household head	Flood I	23	82	42.64	13.66
	Flood II	22	75	43.51	12.60
Household size	Flood I	1	26	7.82	5.13
	Flood II	1	22	8.14	4.00
Annual expenditures on clothing and footwear (FCFA)	Flood I	30 000	600 000	198 690	150 100
	Flood II	5000	250 000	78 380	68 850
Number of groups to which a member of the household belong	Flood I	0	5	2.13	1.47
	Flood II	0	6	1.34	1.31

Note: Currency values have been rounded up to the nearest existing value; 1 US$ ≈FCFA 600.

FCFA, Franc de la Communauté Financière d’Afrique.

The mean sample age of victims (≈43 years) suggests that most victims are still very active. This mean is lower than the life expectancy of 57 years in Cameroon (World Bank 2014b). The mean sample household size (≈8) mirrors other research findings reported for other rural areas in the region (e.g. Ngwa et al. 2015). Large household sizes suggest the importance that flood victims may attach to family labour, a key input in subsistence-based agricultural systems. Such contentions have been raised in previous research works in rural Cameroon (Balgah et al. 2016). The mean annual household expenditure on clothing and footwear for the entire sample of FCFA (Franc de la Communauté Financière d’Afrique) 138 535 (≈$230.00) may be indicative of the importance of dressing in the communities. These expenditures for Flood I victims more than double that of Flood II victims (FCFA 198 690 [≈$330.00] and FCFA 78 380 [≈$130.00], respectively). A possible explanation for this large difference is the fact that Flood I (Babessi) is the seat of the local Council. Households probably have social pressure from non-indigenes to spend more on dressing than in the Flood II (Baba I) community. However, from a hazard management perspective, such expenditures absorb income that could have been available for possible investment in ex ante or ex post-hazard management strategies and demonstrates a likely preference, for instance, for leisure over formal insurance. This conjecture is corroborated by the fact that no household in the two research communities had a flood insurance policy. It is also likely that expenses on clothing and footwear were necessary to command social respect in the community. Further research will be needed to make conclusive statements.

**FIGURE 2:**
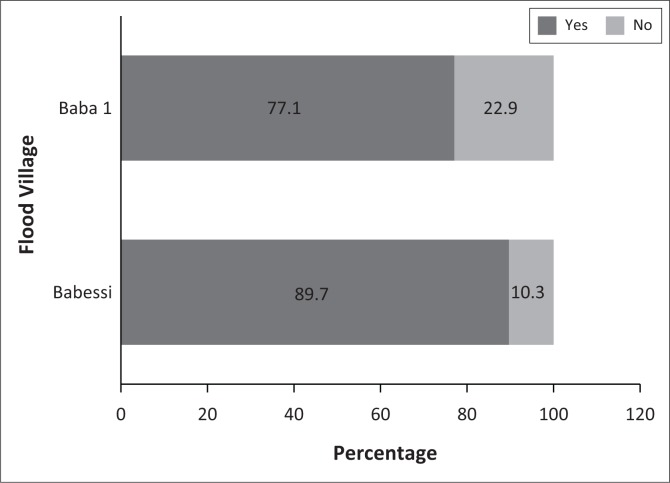
Household membership in groups and/or networks.

**FIGURE 3:**
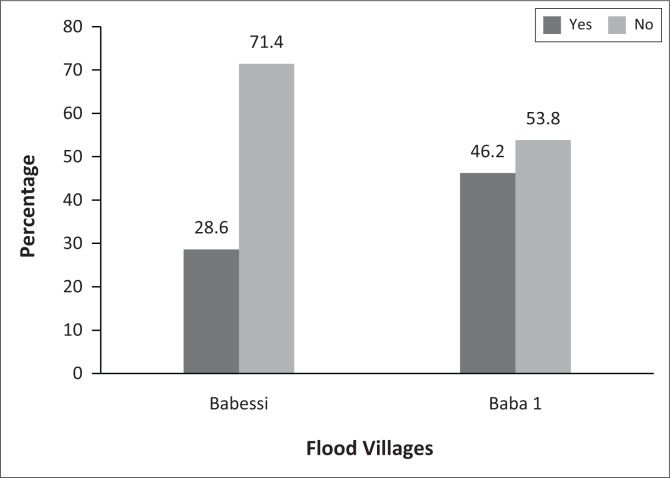
Households with leadership positions in groups or networks.

In general, over 80% of all sampled flood victims (≈90% of Flood I and ≈80% of Flood II) belonged to a group or network. This is shown in Figure 2. However, Flood I households belonged to one more group and/or network as compared to their Flood II counterparts. In addition, 28.6% and 46.2% of the victims from Flood I and II, respectively, reported holding leadership positions in at least one of the groups or networks they belonged to (see Figure 3). These statistics suggest the abundance of social capital, its potential role in hazard coping and its possible influence on household decisions to adopt specific coping strategies after the floods, especially under prevailing conditions of state and market failure. Such contentions have been previously emphasised (Armah et al. 2010; Bastagli & Holmes 2014; Jakiela & Ozier 2012).

### Determinants for adopting informal coping strategies

Descriptive statistics revealed that in general, over 80% of all floods victims applied only informal strategies to cope with the aftermaths of both floods whilst 20% employed a combination of formal and informal instruments to cope with the floods. No household applied solely formal (state and market) coping strategies. Therefore, we attempt to examine which factors were responsible for such biased coping choices observed in two independent floods. Binary logistic regressions were performed to assess the influence of different variables on the households’ decision to predominantly adopt informal coping mechanisms in both case study floods. For a better apprehension, the variables were grouped into human, social and financial or economic capital determinants for the analysis. The grouped variables were separately regressed against a binary variable, namely the household decision to predominantly adopt informal coping strategies after two independent floods. The binary variable was represented as 1, when the household’s coping strategy was informal and 0 when it was not.

The regression model can therefore be represented as:

$$Y=\alpha +\beta_{1}X_{1}+\beta_{2}X_{2}+\ldots +\beta nXn+e$$ [Eqn 1]

where:

*Y* is a binary variable (1,0), indicating the coping decision of the household,

α is the constant,

*β*s the predictors,

*X*1-*X*n are the independent variables influencing the adoption decisions, and e is the error term.

Before doing the analysis by form of capital, the independent variables were grouped and used to establish the goodness-of-fit for each case study flood. Table 2 presents the model output. The results indicate that the independent variables acceptably explain the decision to adopt mainly informal coping strategies in Flood I (*X*^2^≤ 0.05) and not necessarily with the same vigour in Flood II (*X*^2^ ≥ 0.05). As evident from Table 3, almost 52% of the decision to adopt informal coping strategies is explained by the selected variables in Flood I. However, the variables in Flood II account for only slightly above 25% of the decision-making process. Probably, a different set of variables would be needed to convincingly explain the choice of decisions in Flood II. This discrepancy suggests that different variable sets can influence the decision to adopt specific risk-coping strategies even for floods that occur in the same agroecological zone. It is also possible that the combination of different (formal and informal) coping strategies that was not considered separately could be responsible for lowered model performance. However, for the sake of consistency, we had to compare the same variables across the two case study floods. Nevertheless, we generally acknowledge that additional variables other than those tested in our case study would be needed to better explain the decisions in the second case study flood. This of course would require further research.

**TABLE 2:** Omnibus tests of model coefficients.

Regression type	Village	Chi-square	df	Sig.
Step	Flood I	15.680	8	0.05
	Flood II	5.780	8	0.67
Block	Flood I	15.680	8	0.05
	Flood II	5.780	8	0.67
Model	Flood I	15.680	8	0.05
	Flood II	5.780	8	0.67

df, degrees of freedom; Sig., significance.

**TABLE 3:** Model summary.

Community	-2 Log likelihood	Cox & Snell *R* square	Nagelkerke *R* square
Flood I	23.899	0.33	0.52
Flood II	26.290	0.15	0.25

Note: Estimation was terminated at iteration number 7, as parameter estimates changed by less than 0.001.

Furthermore, we examined the robustness of similar important determinants in influencing coping strategies for both floods. In our opinion, the robustness of variables across case studies will provide support for their consideration in flood-related policy prescriptions of wider (national or regional) relevance.

#### Human capital

Regression results for human capital variables are presented in Table 4. Human capital variables largely influenced coping decisions in the two flood case studies differently. With the exception of household size, all the other variables positively influenced the coping decisions of Flood I victims, as observed from the beta values. These results support existing contentions from independent, cross-sectional studies (Balgah et al. 2016; Jensen 2012; Mobarak et al. 2012). However, the exact opposite effect was observed in Flood II. As mentioned earlier, the variables tested in Flood II explain only 25% of the flood-coping decisions. This in effect means that there are other important determinants, which were not captured in our research. Further research will be necessary to provide better explanations to these observations. Nevertheless, such results draw our attention to the fact that coping determinants can be quite different even for floods occurring in the same agroecological zone. As suggested by Boamah et al. (2015), whilst an individual case study approach is useful, solid trends on which broad-based (flood) policies can depend will only emerge from the analysis of multiple cases across space and time.

**TABLE 4:** Human capital variables.

Variable	Village	*β*	S.E.	Wald	Df	Sig.	Exp(*β*)
Age	Flood I	0.080	0.076	1.099	1	0.30	1.083
	Flood II	−0.033	0.064	0.262	1	0.61	0.968
Education	Flood I	0.464	0.495	0.879	1	0.35	1.591
	Flood II	−1.082	0.744	2.118	1	0.15	0.339
Household size	Flood I	−0.024	0.159	0.022	1	0.88	0.977
	Flood II	−0.150	0.162	0.860	1	0.35	0.860
Main occupation of household head	Flood I	2.900	1.850	2.457	1	0.12	18.171
	Flood II	−0.010	1.111	0.000	1	0.99	0.990

*β*, beta value; S.E., standard error; df, degrees of freedom; Wald, Wald test of true parameter value; Exp(*β*), expected beta value; Sig., significance.

#### Social capital

Table 5 presents the results of the regression analysis on social capital variables. Marital status and membership in groups or networks positively influenced Flood I victims to mainly adopt informal coping strategies, with the latter being significant at the 5% level (*p* = 0.009). With the exception of leadership positions held in groups and networks (and the above mentioned), all other tested social capital variables had negative values in both case study floods. Belonging to a group or network did not automatically translate into benefiting from these networks after the floods, as generally conjectured in the topical literature (Grotaert et al. 2004). This suggests that the type of groups and networks matter. It had to take leadership and therefore additional influence in these networks, for instance, for Flood I victims to benefit from the abundance of social capital captured through membership in networks. Even the gender of the household head did not demonstrate a positive influence on the coping decisions in both case studies, suggesting that female-headed households were more likely to adopt informal coping strategies than male-headed ones. The results generally deviate from contemporary positions in the topical state of the art on the relevance of social capital for risk-coping in developing countries (Armah et al. 2010; Balgah et al. 2016; Bastagli & Holmes 2014; Jakiela & Ozier 2012; Mobarak et al. 2012). However, they reiterates the fact that the type of social capital is crucial for it to significantly influence coping decisions of flood victims. However, further research is required to draw firmer conclusions.

**TABLE 5:** The level to which social capital variables influence coping strategies by floods.

Variable	Community	*β*	S.E.	Wald	df	Sig.	Exp(*β*)
Marital status	Flood I	3.142	1.635	3.694	1	0.050	23.145
	Flood II	−0.299	0.878	0.116	1	0.730	0.742
Membership in groups or networks	Flood I	6.557	2.511	6.821	1	0.010	703.990
	Flood II	−1.273	1.636	0.606	1	0.440	0.280
Leadership in groups or networks	Flood I	−0.921	1.206	0.583	1	0.450	0.398
	Flood II	0.948	1.299	0.532	1	0.470	2.579
Gender	Flood I	−3.088	1.764	3.064	1	0.080	0.046
	Flood II	−1.527	1.455	1.101	1	0.290	0.217

*β*, beta value; S.E., standard error; df, degrees of freedom; Wald, Wald test of true parameter value; Exp(*β*), expected beta value; Sig., significance.

#### Financial and economic capitals

Following Zeller et al. (2006), financial capital was captured mainly through the estimated household income at the time of the floods, whilst economic capital was computed as the total value of some selected household assets and the livestock owned by the household at the time of the floods. The summary results are presented in Table 6. The results clearly show that economic and financial capitals had no influence on flood victims’ coping decisions. These results widely deviate from the general scholarly contentions, where economic and financial capitals have been identified as key determinants for coping with natural hazards (see, for instance, Cervantes-Godoy et al. 2013; Holzmann 2001; Meredith et al. 2013). It departs even from simple logic! We explain these rather strange findings that financial and economic capitals may have been normally distributed amongst flood victims, most of whom are generally likely to be living in poverty. As such, financial and economic capitals could not have been robust enough to determine coping decisions in the research region, because households cannot be substantially differentiated based on these variables. Such contentions have been raised in recent research in the region (e.g. Balgah et al. 2016). However, further research is necessary to draw firmer conclusions.

**TABLE 6:** Analysis of economic and financial capital by flood type.

Village	Variable	*β*	S.E.	Wald	df	Sig.	Exp(*β*)
Flood I	Economic capital	0.000	0.000	0.941	1	0.33	1.000
	Financial capital	0.000	0.000	0.148	1	0.70	1.000
Flood II	Economic capital	0.000	0.000	1.408	1	0.24	1.000
	Financial capital	0.000	0.000	2.527	1	0.11	1.000

*β*, beta value; S.E., standard error; df, degrees of freedom; Wald, test of true parameter value; Exp(*β*), expected beta value; Sig., significance.

## Conclusion and recommendations

This paper has assessed the different drivers for household decisions to flood hazards, based on two case study floods in Cameroon. The objective was to identify drivers that go beyond individual, isolated case studies currently dominating the topical literature and identify potential determinants robust across multiple floods, which could be appropriated for flood hazard policy making. The study focused on two neighbouring villages (case study sites) in the Northwest Region of Cameroon that are prone to frequent flooding. Empirical research involving quantitative and qualitative data were collected and analysed from flood-affected households in the two case study floods. Descriptive statistics and regression analyses results were used to test the robustness of selected human, social, and economic or financial variables in influencing household coping decisions across the two case studies. Same variables were used for both case studies to permit comparability. The findings have revealed mixed results – human capital variables, for instance, have been found to positively affect household coping decisions in Flood I case study and not in Flood II. A differentiated relevance of social capital was also observed in both study sites. Whilst group membership and social networks were crucial for coping decision-making in Flood I, holding leadership position in these groups was more decisive in Flood II. Financial and economic capitals had no influence on coping decisions in both case studies. Overall, the tested variables explained only 52% and 25% of the total coping decisions for case study one and two, respectively. This, we assume, is partially because around 20% of all victims used a combination of informal (community-based) and formal (state led) strategies to cope with the floods. In addition, it is also possible that the initially selected variables in our model (based on the contemporary state of the art) did not include the key variables that were specifically relevant for the flood victims. However, this was necessary for comparative analysis across the two case study floods.

Given the current status quo, it seems plausible to hypothesise that flood victims in developing countries are likely to depend heavily on informal coping strategies as a result of widespread state and market failures for flood risk management. However, given the opportunity, they would appropriate formal instruments and opportunities to cope with flood hazards. In the meantime, research should continue to support policymakers by increasingly analysing multiple case studies so that trends can be identified to support policy decisions for flood risk management beyond individual case studies. A starting point could be to undertake an in-depth review of the existing literature and cautiously identify robust determinants across published case studies. This should be accompanied by increased risk reduction measures that can mitigate the impacts of floods when they occur. Perhaps, developing countries should take the prevention and preparedness for flood risks more seriously, as they stand to suffer most from increasing flood hazards, predominantly because of the failure of state and market institutions for flood hazard management.
